# A Pocket Medical Dictionary

**Published:** 1900-06

**Authors:** 


A Pocket Medical Dictionary.—Giving the pronunciation and definition of the principal words used in medicine and the collateral sciences, including very complete tables of clinical eponymic terms of the arteries, muscles, nerves, bacteria, bacilli, micrococci, spirilla and thermometric scales, and a dose-list of drugs and their preparations, in both the English and metric systems of weights and measures. By George M. Gould, A. M., M. D., author of "The Illustrated Medical Dictionary," "The Student's Medical Dictionary," editor of the *Philadelphia Medical Journal*, etc. Fourth edition; enlarged and revised; 30,000 words. Philadelphia: P. Blakiston's Son & Co., 1012 Walnut Street. 1900. Price, $1.00.

This is an ideal pocket dictionary for medical men and students. It will fit anybody's pocket, the book is flexible, the type is clear, and it is remarkable for the amount of literary material contained between its covers. It gives the same words and pronunciations as the author's larger dictionaries give, and it only costs a dollar.

